# Ciprofloxacin-induced Stevens-Johnson Syndrome with Grapefruit Juice Consumption: A Case Report

**DOI:** 10.7759/cureus.3827

**Published:** 2019-01-04

**Authors:** Matthew G Cravens, Nathan Sherman, Jennifer Sawaya

**Affiliations:** 1 Emergency Medicine, University of Arizona College of Medicine, Tucson, USA; 2 Internal Medicine, University of Arizona College of Medicine, Tucson, USA; 3 Dermatology, University of Arizona College of Medicine, Tucson, USA

**Keywords:** stevens-johnson syndrome, ciprofloxacin, sjs, cytochrome p450

## Abstract

We describe the case of a 49-year-old, otherwise healthy, Hispanic male who underwent an uncomplicated vasectomy and was treated prophylactically with a one-week course of ciprofloxacin. Two days after completing the antibiotic course, he developed a pruritic, blistering rash that covered 90% of his body surface area. Punch biopsy of the skin lesions confirmed the diagnosis of Stevens-Johnson syndrome (SJS). Upon further questioning, it was revealed that the patient had consumed approximately 32 ounces of grapefruit juice each of the seven days following his vasectomy. We hypothesized that the cytochrome P450 inhibitory effect of grapefruit juice had dramatically elevated systemic levels of ciprofloxacin, increasing the risk of developing SJS. Literature review revealed that ciprofloxacin is metabolized primarily by CYP1A2 with partial CYP3A4 metabolism, while grapefruit juice is strictly an enterocyte CYP3A4 inhibitor. To the authors’ knowledge, consumption of grapefruit juice has never been demonstrated to increase systemic levels of ciprofloxacin or of other fluoroquinolones. We conclude that either this is the first reported case of a grapefruit juice-ciprofloxacin interaction causing SJS, or that this is simply ciprofloxacin-induced SJS. Importantly, ciprofloxacin is not recommended by the American Urological Association for a routine vasectomy without risk factors for infection. We remind clinicians that inappropriately prescribed antibiotic prophylaxis for routine procedures can cause serious morbidity, including SJS, and should only be prescribed when indicated.

## Introduction

Stevens-Johnson syndrome (SJS) is a severe, desquamating disease most commonly caused by prescription medications. Ciprofloxacin is a rare but an established cause of SJS [1]. Herein, we describe a case of SJS assumed to be associated with ciprofloxacin prophylaxis following an uncomplicated vasectomy. The patient consumed large quantities of grapefruit juice concurrent with his antibiotic therapy. As SJS can be a dose-dependent phenomenon [2], the authors hypothesized that the inhibitory effects of grapefruit juice on cytochrome P450 resulted in extreme circulating levels of ciprofloxacin and thus increased his risk of SJS.

## Case presentation

**Figure 1:**
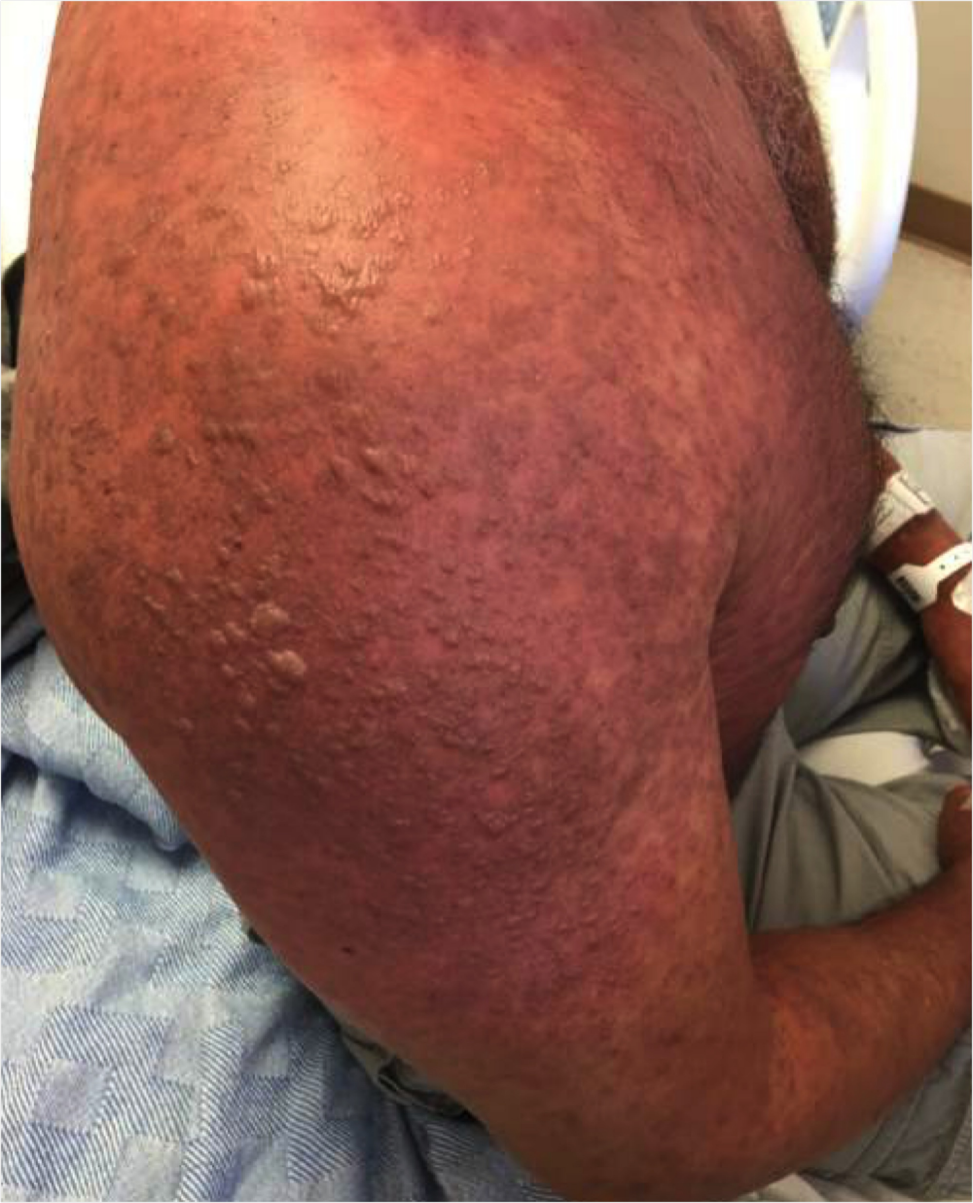
Patient after two days of hospitalization Note: the bullae demonstrating epidermal separation, consistent with Stevens-Johnson syndrome

A 49-year-old, otherwise healthy, Hispanic male underwent an uncomplicated vasectomy and was discharged with a one-week supply of standard-dose ibuprofen and ciprofloxacin. He denied taking other medications or supplements prior to the procedure. Two days after completing his ciprofloxacin regimen, a pruritic, maculopapular rash began spreading progressively from his posterior neck to the face, trunk, and all extremities over five days. At presentation, blistering and desquamation had developed globally; however, the soles of the feet and hair-bearing skin of the head were spared, as was a circumorbital distribution extending posteriorly across the sides of his face, which corresponded to the sun-shielded areas associated with his regular use of sunglasses. Mild mucosal ulceration of the mouth and eyelids was present, and an estimated 90% of the skin was involved, though with less than 10% epidermal detachment (Figure 1). Punch biopsies confirmed the diagnosis of SJS. Careful history revealed that the patient had consumed approximately 32 ounces of grapefruit juice with 2 to 4 servings of alcohol every evening for seven days following his vasectomy. He reported this large consumption of grapefruit juice and alcohol as abnormal for him.

## Discussion

Ciprofloxacin is an established but a rare cause of SJS [1]. Concomitant ciprofloxacin and grapefruit juice consumption leading to SJS, however, has never been documented. Initially, there was concern that the cytochrome inhibitory effect of grapefruit juice had dramatically increased serum levels of ciprofloxacin. As drug-induced SJS may be dose-dependent [2], we hypothesized that the patient had extreme levels of ciprofloxacin, causing SJS. 

An extensive literature review did not reveal any similar cases but did highlight the importance of understanding the basic science behind drug interactions. Grapefruit juice increases the serum levels of some drugs by irreversibly inhibiting CYP3A4 enzymes in enterocytes, preventing drug metabolism at the gut level prior to systemic distribution. It also inhibits the efflux of some drugs back into the gut lumen, by blocking P-glycoprotein. Grapefruit juice’s effect seems almost completely limited to the gut, such that intravenous administration would have little to no effect [3]. Ciprofloxacin is metabolized primarily by CYP1A2, and though it has some CYP3A4 metabolism, there is no evidence in the literature that ciprofloxacin or other fluoroquinolones are increased by grapefruit juice consumption. Yet, as ciprofloxacin is partially metabolized by CYP3A4, it is possible that this is the first reported case of a clinically significant interaction with grapefruit juice.

We conclude that this is either the first reported case of a grapefruit juice-ciprofloxacin interaction causing SJS or simply SJS caused by prophylactic administration of ciprofloxacin following vasectomy. Importantly, the American Urological Association guidelines do not recommend prophylactic antibiotics for a routine vasectomy without risk factors for infection [4]. This case is thus a compelling reminder of the risks associated with prescribing antibiotics, as this patient’s morbid condition appears to have directly resulted from an antibiotic that was not indicated for his routine procedure. 

Finally, while this case is strongly temporally related to ciprofloxacin administration, other causes of SJS must be considered. First, the nonsteroidal anti-inflammatory drugs are not uncommon causes of SJS, and it is possible that ibuprofen alone, an ibuprofen-ciprofloxacin interaction, an ibuprofen-grapefruit juice interaction, or a combination thereof was responsible for this case. We felt that the patient's likely prior exposure to ibuprofen and the lack of history of exposure to ciprofloxacin were sufficient to exclude this possibility for the purpose of this discussion. Non-pharmacologic causes are also a possibility. An undiagnosed infection, classically with *Mycoplasma pneumoniae*, could have induced the patient's presentation. Numerous other causes of SJS have been proposed, and in over one-third of SJS cases, no cause can be identified [5]. We hope that while causality is difficult to prove in this case, valuable information is gained from this discussion regarding CYP metabolism and the risks of prescribing non-indicated antibiotics.

## Conclusions

Ciprofloxacin is an established but a rare cause of SJS and is metabolized primarily by CYP1A2 with partial CYP3A4 metabolism. Grapefruit juice is a CYP3A4 inhibitor in the gut only. This case consists of drug-induced SJS thought to be complicated by grapefruit juice consumption. However, no evidence was found in the literature of grapefruit juice increasing levels of fluoroquinolones. We conclude that this is likely either the first case of grapefruit juice-ciprofloxacin interaction causing SJS or simply ciprofloxacin-induced SJS. Additionally, by American Urological Society guidelines, ciprofloxacin was not indicated for this vasectomy patient without risk factors for infection. Prophylactic antibiotics for routine procedures can cause serious morbidity including SJS and should only be prescribed when indicated.
